# Debate: What guidance is needed by academics who collaborate with digital companies to improve youth mental health?

**DOI:** 10.1111/camh.12779

**Published:** 2025-04-22

**Authors:** Jake Bourgaize, Jacob Andrews, Camilla Babbage, Marianne E. Etherson, Joanne Gregory, Chris Hollis, Kareem Khan, Sieun Lee, Joanna Lockwood, Josimar Mendes, Jen Martin, Emma Nielsen, Adam Parker, Ellen Townsend, A. Jess Williams, Rebecca Woodcock, Sonia Livingstone, the Digital Youth Research Programme

**Affiliations:** ^1^ Department of Child and Adolescent Psychiatry King's College London London UK; ^2^ NIHR HealthTech Research Centre for Mental Health, University of Nottingham Nottingham UK; ^3^ Academic Unit of Mental Health and Clinical Neuroscience, School of Medicine University of Nottingham Nottingham UK; ^4^ School of Health & Wellbeing, University of Glasgow Glasgow UK; ^5^ Beacons of Excellence, University of Nottingham Nottingham UK; ^6^ School of Applied Social Sciences, De Montfort University Leicester UK; ^7^ Department of Computer Science University of Oxford Oxford UK; ^8^ School of Psychology, University of Nottingham Nottingham UK; ^9^ Department of Informatics King's College London London UK; ^10^ Department of Media and Communications London School of Economics and Political Science London UK

**Keywords:** Debate, digital, technology, industry, psychological methods, children, adolescent, social media, digital media, mental health

## Abstract

In a recent debate piece, Livingstone, Orben and Odgers (*Child and Adolescent Mental Health*, 2023, 28, 150) asked whether and when it is advisable for academics researching youth mental health to collaborate with digital companies. Such collaborations arise when researchers seek to identify risks and opportunities associated with youth digital engagement or to develop research‐based interventions that leverage digital technologies to improve young people's mental health. However, young people's digital experiences, the associated data records and the prospects of testing interventions in situ are often under the control of digital providers and inaccessible to researchers. There is growing optimism that collaboration with companies may allow independent researchers access to proprietary resources along with opportunities to test and scale up beneficial digital interventions. Understandably, such optimism is tempered by scepticism about the processes, trust, and interests at stake during collaboration between researchers and companies. Given these often opposed positions, it is clear that professional guidance is required for academics so they understand the potential costs and benefits of choosing to enter into such collaborations and the terms on which this is advisable. This article highlights what information already exists for researchers and what guidance is needed to ensure that youth mental health researchers can successfully collaborate with digital companies.

‘Digital’ can refer to any non‐physical content (e.g. music, images, videos, games or text) created, stored or transmitted electronically. It also refers to the companies that create, host and manage such content. In the digital age, research cannot expect fully to understand adolescents' mental health without recognising the impact of this content (and related companies) on young people's lives. Hollis, Livingstone, and Sonuga‐Barke (2020) observe that, on the one hand, digital technology brings opportunities for youth participation, escape, support and help, but on the other hand, it poses risks of harm that amplify or compound prior mental health difficulties or even introduce new ones. While such technology affords innovative and efficient prospects for mental health interventions to benefit young people, researching young people's interactions with it is difficult. Many digital activities, the data traces that record them, and the automated processing and design features that shape youth experiences are inaccessible to researchers, though they are of course accessible to (and used by) digital platforms, data brokers and other commercial actors.

In a recent debate in *Child and Adolescent Mental Health*, Livingstone, Orben, and Odgers (2023) asked whether and when it is advisable for academics researching youth mental health to collaborate with digital companies. As the responses revealed, arguments can become polarised between those who defend principles of intellectual independence and research ethics versus those who advocate the necessity of cooperation for practical impact. There is some optimism regarding productive collaboration when academic, public and commercial interests align (more likely in researching the benefits of digital technology). But there is deep scepticism that conflicts of interest may undermine robust, unbiased research (more likely if the research concerns risks or interventions). In a field where few digitally based mental health innovations are both supported by research evidence and widely available, we urge that this professional community finds a way forward.

In principle, collaboration with digital companies can provide access to valuable data, sharing professional and technical expertise, real‐time in situ hypothesis testing and rolling out (managing, investing in, delivering) therapeutic interventions to a professional standard and at scale. Collaboration is already widely promoted by university accelerators, funding partnerships and mandated pathways to impact. Regulators are seeking legal means to require companies to share proprietary data with researchers to serve the public interest. While we are not of the view that such collaborations are advisable in all instances, we ask the following: what guidance is needed for academic and clinical researchers who do decide to work with industry partners?

## Professional guidance is needed

Over the past year, members of the UKRI‐funded Digital Youth programme worked together to identify the challenges and propose practical ways ahead. During our discussions, it appeared that both early career and senior researchers want guidance on how to decide whether to collaborate with digital companies, whom to consult, what conditions to set, the pitfalls to avoid, and what good practice exists. We also learned that, at present, these questions are seldom prioritised formally – such as through university or funder guidance, ethics review boards or academic journal requirements. Nor, we believe, are they much discussed during mentoring, professional training or peer exchanges.

One risk is that ill‐informed or haphazard decisions are made or, at worst, that research integrity is compromised, in turn risking public trust. Another is that opportunities are missed to embed high standards of research integrity in professional training programmes. A third is that, insofar as decisions about research collaboration are left to individuals, the academy loses the wider benefit for the collective or public good of negotiating terms with big business from a position of power.

Of course, some protections are already in place. Academic research requires institutional ethics approval to safeguard participants' well‐being. The legal departments of academic and commercial partners will write contracts that protect data sharing and intellectual property, among other considerations. Even so, as research collaborations and open science become more the norm, it is worth examining whether emerging practices are sufficiently addressed and training is updated.

The Digital Youth group began reading ethical codes of practice, contract guidance from our universities, and professional documentation. We also asked colleagues who had advised them how they reached their decisions and what guidance would be helpful. We explored analogous situations – for example, where guidance exists, it is often derived from collaborations with tobacco companies or pharmaceutical industries.

## Forewarned is forearmed

What guidance would be useful? Fully answering this question will require research, and professional discussion and co‐design in its own right. To begin this process, we offer the following thoughts from our Digital Youth discussions. The above commentary has already addressed a fundamental question, namely the purpose of the proposed research collaboration. The purpose may be regarded differently by the academic and commercial partners, and there is clear value in ascertaining anticipated benefits and risks of the research – for public knowledge (or, for youth in particular) and, also, for the partners. Just asking what each side stands to gain or lose can be illuminating.

Then, it is important to conduct due diligence checks on the potential collaborator. These could usefully include questions such as the following:

- Has the company signed up to principles of research ethics and good practice (e.g. Responsible Research Innovation, Ethical Artificial Intelligence, Certified B Corporation or National Health Service Digital Technology Assessment Criteria)?
- Does the company's track record indicate openness to working with service/end users (patient and public involvement)?
- Does the company have public/service users on its governance or oversight boards or support an effective youth panel?
- Does the company compensate (young) service users for their time in patient and public involvement activities?

Other questions may be difficult to ask directly of the potential collaborator. Crucially, the researcher needs to identify if there are reasons why the collaboration may be avoided. This could be where the company has a documented history of impeding academic independence in industry‐funded research (e.g. Goldberg & Vandenberg, 2021) or including conditional clauses in collaboration contracts (e.g. allowing companies the right to withhold publication rights following data analysis; Mello et al., 2018). This is particularly important for early‐career researchers at risk of being manipulated by digital companies due to their greater need for funding and publications for career advancement.

Thus far, we have found little documentation of feared risks (e.g. co‐option or manipulation of intellectual integrity or research findings). But in other domains, researchers have documented the potentially detrimental impact of industry sponsorship – consider food and tobacco (Hong & Bero, 2002) research. For researchers, the rising number of whistleblower accounts from social media platforms regarding youth mental health research does not bode well here.

Less visible is the potential for chilling effects, where researchers may self‐limit the scope or reporting or impact of their research, whether out of fear or ignorance or hope of gain. This raises the further question – at what stage of the research should the collaboration begin: some consider sponsor involvement in framing the research questions inappropriate, for instance Hong and Bero (2002), while others consider this usual. More attention has been paid to which findings are reported and how, though again, guidance regarding the involvement of a corporate partner is lacking. We note Coco Cola's insistence to retain the rights to (not) publish research results or terminate ongoing research projects without reason (see Mahase (2019) for the (non‐digital) case of Coca‐Cola's academic collaboration research clauses).

Even when there is no problematic practice, and the research collaboration goes smoothly, researchers need guidance to protect their reputation for integrity. For example, following their collaboration with Meta, Vuorre and Przybylski (2023) reported that analysis of their dataset of 946,798 Meta product users suggested no relationship between the spread of social media and psychological harm. These results were met with scepticism by the then Head of Child Safety Online Policy at the NSPCC: ‘The latest example of Meta giving favoured researchers preferential access to data sets to skew the evidence base’ Burrows (2023).

## Guidance can identify benefits as well as risks

Just as some of the risks are hard to identify or document, we note that, in a context where significant concerns exist regarding the potential problems of collaboration with digital companies (Livingstone et al., 2023; Shotbolt, 2023), it is hard to point to clear accounts of the benefits – where collaboration with such companies has led to valuable and otherwise unobtainable insights. As we have further argued, it is also difficult to find convincing accounts of the conditions under which successful collaborations with digital companies have been undertaken. Nor does the literature on youth mental health in digital contexts carefully document what can, and what has, gone wrong, so that the research community can learn from this experience.

Documentation of successful collaborations would be very helpful to guide other researchers in future collaborations with digital companies. For example, one instance of good practice within our team was formalising contractual agreements regarding data sharing and intellectual property in writing, before the collaboration began, and in such a way as to protect both researchers and research participants. Helpful guidance is provided by the World Health Organization (2017) Code of Conduct for Responsible Research, particularly surrounding data sharing and intellectual property. Formalising such agreements prior to collaboration creates transparency that allows for academics to make an informed decision before entering said collaborations. Nonetheless, alternative frameworks may also be advisable.

At present, we contend that clear guidance for industry collaboration is hard to find for researchers of child and adolescent mental health. Nor do we know if such guidance is sufficient or effective. For the good of our professional community as well as the young people whom our research should ultimately benefit, we encourage our readers to consider what next steps should be undertaken to produce the guidance needed.

## Funding information

The authors (JB, JA, CB, ME, JG, CH, KK, SL, JL, JM, JM, EN, AP, ET, JW, RW and SL) acknowledge the support of the UK Research and Innovation (UKRI) Digital Youth Programme award [MRC project reference MR/W002450/1] which is part of the AHRC/ESRC/MRC Adolescence, Mental Health and the Developing Mind programme and Sprouting Minds. JA receives support from the Office for Life Sciences and the National Institute for Health and Care Research (NIHR) Mental Health Translational Research Collaboration, hosted by the NIHR Oxford Health Biomedical Research Centre.

## Conflict of interest statement

The authors have declared that they have no competing or potential conflicts of interest.

## Ethics statement

No ethical approval was required for this debate article.

## Data Availability

Data sharing is not applicable to this article as no new data were created or analysed.
